# Correction: microRNA-613 exerts anti-angiogenic effect on nasopharyngeal carcinoma cells through inactivating the AKT signaling pathway by downregulating FN1

**DOI:** 10.1042/BSR-20182196_COR

**Published:** 2020-12-10

**Keywords:** AKT signaling pathway, FN1, Invasion, MicroRNA-613, Migration, Nasopharyngeal carcinoma

This Correction follows an Expression of Concern relating to this article previously published by Portland Press.

The authors of the original article above article “microRNA-613 exerts anti-angiogenic effect on nasopharyngeal carcinoma cells through inactivating the AKT signaling pathway by downregulating FN1” (Biosci Rep (2019) 39(7), 10.1042/BSR20182196) would like to correct Figure 3D. Due to the nonstandardised naming of pictures stored in transwell experiment and an error in the authors’ figure creation, an incorrect image was selected for the blank group. The authors declare that these corrections do not change the results or conclusions of their paper, and express their sincere apologies for any inconvenience that this error has caused to the readers. The corrected version of Figure 3 is presented here.

**Figure 3:**
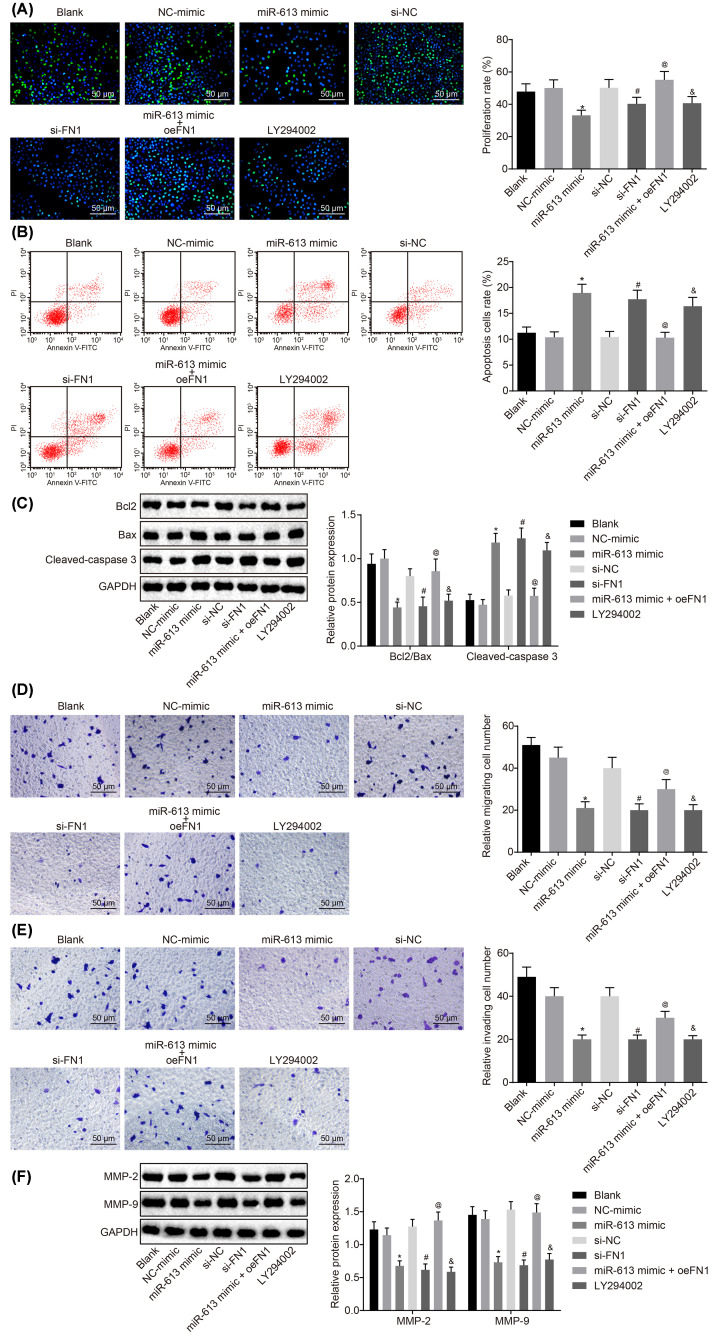
Up-regulated miR-613 or down-regulated FN1 contributes to suppressed cell proliferation, invasion, and migration, and promoted cell apoptosis of HONE1 cells (**A**) Cell proliferation of HONE1 cells after treatments with the mimic or inhibitor of miR-613 and FN1 as well as the LY294002 treatment detected by EdU (200×); (**B**) cell apoptosis of HONE1 cells after treatments with the mimic or inhibitor of miR-613 and FN1 as well as the LY294002 treatment examined by flow cytometry; (**C**) the protein bands of Bcl-2, Bax, Cleaved-caspase3 in HONE1 cells examined by Western blot analysis; (**D**) cell migration of HONE1 cells after treatments with the mimic or inhibitor of miR-613 and FN1 as well as the LY294002 treatment detected by Transwell assay (200×); (**E**) cell invasion of HONE1 cells after treatments with the mimic or inhibitor of miR-613 and FN1 as well as the LY294002 treatment detected by Transwell assay (200×); (**F**) protein expression of MMP-2 and MMP-9 in HONE1 cells after treatments with the mimic or inhibitor of miR-613 and FN1 as well as the LY294002 treatment examined by Western blot analysis. ^&^*P*<0.05 compared with the blank group; *P<0.05 compared with the NC mimic group; ^#^*P*<0.05 compared with the si-NC group; ^@^*P*<0.05 compared with the miR-613 mimic group; the measurement data were expressed as the mean ± standard deviation; data among multiple groups were compared by one-way ANOVA; the experiment was repeated three times.

